# Replicability of structural brain alterations associated with general psychopathology: evidence from a population-representative birth cohort

**DOI:** 10.1038/s41380-019-0621-z

**Published:** 2019-12-03

**Authors:** Adrienne L. Romer, Annchen R. Knodt, Maria L. Sison, David Ireland, Renate Houts, Sandhya Ramrakha, Richie Poulton, Ross Keenan, Tracy R. Melzer, Terrie E. Moffitt, Avshalom Caspi, Ahmad R. Hariri

**Affiliations:** 1 Laboratory of NeuroGenetics, Duke University, Durham, NC USA; 2 Department of Psychology & Neuroscience, Duke University, Durham, NC USA; 3 Dunedin Multidisciplinary Health and Development Research Unit, Department of Psychology, University of Otago, Dunedin, New Zealand; 4 Christchurch Radiology Group, Christchurch, New Zealand; 5 Department of Medicine, University of Otago, Christchurch, New Zealand; 6 New Zealand Brain Research Institute, Christchurch, New Zealand; 7 Department of Psychiatry & Behavioral Sciences, Duke University School of Medicine, Durham, NC USA; 8 Center for Genomic and Computational Biology, Duke University, Durham, NC USA; 9 Social, Genetic, and Developmental Psychiatry Research Center, Institute of Psychiatry, Psychology & Neuroscience, King’s College, London, England

**Keywords:** Psychiatric disorders, Biomarkers, Neuroscience, Psychology

## Abstract

Transdiagnostic research has identified a general psychopathology factor—often called the ‘*p*’ factor—that accounts for shared variation across internalizing, externalizing, and thought disorders in diverse samples. It has been argued that the *p* factor may reflect dysfunctional thinking present in serious mental illness. In support of this, we previously used a theory-free, data-driven multimodal neuroimaging approach to find that higher *p* factor scores are associated with structural alterations within a cerebello-thalamo-cortical circuit (CTCC) and visual association cortex, both of which are important for monitoring and coordinating information processing in the service of executive control. Here we attempt to replicate these associations by conducting region-of-interest analyses using data from 875 members of the Dunedin Longitudinal Study, a five-decade study of a population-representative birth cohort, collected when they were 45 years old. We further sought to replicate a more recent report that *p* factor scores can be predicted by patterns of distributed cerebellar morphology as estimated through independent component analysis. We successfully replicated associations between higher *p* factor scores and both reduced gray matter volume of the visual association cortex and fractional anisotropy of pontine white matter pathways within the CTCC. In contrast, we failed to replicate prior associations between cerebellar structure and *p* factor scores. Collectively, our findings encourage further focus on the CTCC and visual association cortex as core neural substrates and potential biomarkers of general psychopathology.

## Introduction

A rapidly emerging body of research has identified a general factor that captures shared variation among multiple forms of psychopathology across diverse samples [1]. This general psychopathology or ‘*p*’ factor [2] accounts for the high rates of comorbidity among internalizing, externalizing, and thought disorders. Multiple explanations of the meaning of the *p* factor have been proposed, including that the *p* factor may index functional impairment, negative affect, emotion dysregulation, and poor intellectual function (for a review see [3]). One compelling argument regarding the nature of the *p* factor is that it captures the extent of disordered or dysfunctional thinking present not only in thought disorders, but also in extreme presentations of internalizing and externalizing disorders [3]. Consistent with this argument, we recently used a theory-free, data-driven approach to find that among 1246 university students higher *p* factor scores were associated with structural alterations in a cerebello-thalamo-cortical circuit (CTCC) critical for monitoring and coordinating information processing in the service of executive control [4].

Specifically, we found that higher *p* factor scores were associated with reduced gray matter volume (GMV) in neocerebellar lobule VIIb. This neocerebellar region is a component of a specific CTCC, including the orbitofrontal, dorsolateral, and medial prefrontal cortices [5, 6]. We also found evidence for decreased microstructural integrity of pontine white matter pathways, as indexed by decreased fractional anisotropy (FA), which mediate communication of information from the prefrontal cortex to the neocerebellum within this CTCC [7–11]. Investigators have theorized that this prefrontal CTCC plays a crucial role in comparing intention with the execution of thoughts, emotions, and actions by continuously updating internal models [12, 13]. Moreover, prefrontal CTCC dysfunction has been consistently reported in disorders principally characterized by poor executive control and disorganized thought such as schizophrenia (e.g. [14, 15]), and individuals with cerebellar cognitive affective syndrome following damage to the neocerebellum experience executive control dysfunction symptoms referred to as “dysmetria of thought” [16–18].

A subsequent report based on analyses of data from 1401 community volunteers revealed that patterns of distributed cerebellar morphology also were associated with general psychopathology as estimated through independent component analysis (ICA) [19]. Namely, morphological features within a cerebellar component involved in cognitive functions (i.e., verbal working memory, retrieval, rehearsal, etc.), as well as reduced GMVs within neocerebellar lobule VI and crus I, were associated with higher general psychopathology. Further, these neocerebellar morphological features were the most important predictors of general psychopathology as compared with 52 other brain-wide anatomical features.

In addition to these structural alterations within neocerebellum and broader prefrontal CTCC, we found novel evidence for decreased GMV in the visual association cortex of individuals with higher *p* factor scores [4]. Subsequently, we found that higher *p* factor scores were associated with patterns of inefficient intrinsic functional connectivity between visual association cortex and networks supporting executive control and self-referential processes, which are implicated across mental disorders [20]. Collectively, these patterns are consistent with speculation that higher *p* factor scores ultimately represent the likelihood of experiencing disordered thought through a diminished capacity for basic monitoring and processing of information supported by the prefrontal CTCC and connectome-wide intrinsic functional connectivity. Such patterns of brain dysfunction may also contribute to negative affect, emotion dysregulation, and inefficient information processing, all of which also have been posited as potential explanations of the *p* factor [3].

It is important to seek to replicate these associations, especially because our original associations were discovered in a convenience sample of high-functioning 18–22-year-old university students through the Duke Neurogenetics Study [4]. Here we attempt to replicate our original associations between prefrontal CTCC and visual association cortex structure and *p* factor scores using data from the Dunedin Longitudinal Study, a five-decade longitudinal study of a population-representative birth cohort now in midlife. Using data from the Dunedin Study, we further sought to replicate the independent components analysis of cerebellar morphology and general psychopathology as reported by Moberget et al. [19] in their study of young community volunteers.

## Materials and methods

### Participants

Participants are members of the Dunedin Study, a longitudinal investigation of health and behavior in a representative birth cohort. Study members (*n* = 1037; 91% of eligible births; 52% male) were all individuals born between April 1972 and March 1973 in Dunedin, New Zealand (NZ), who were eligible based on residence in the province and who participated in the first assessment at age 3 years [21]. The cohort represented the full range of socioeconomic status (SES) in the general population of NZ’s South Island and as adults matched the NZ National Health and Nutrition Survey on key adult health indicators (e.g., body mass index, smoking, GP visits) and the NZ Census of citizens of the same age on educational attainment. The cohort is primarily white (93%), matching South Island demographics [21]. Assessments were carried out at birth and ages 3, 5, 7, 9, 11, 13, 15, 18, 21, 26, 32, 38, and most recently (completed April 2019) 45 years, when 94.1% (*n* = 938) of the 997 participants still alive took part, and 875 (93%) of these age-45 participants also completed MRI scanning (see Supplementary Information, including Supplementary Fig. 1A–C, for further details). Attrition analyses revealed that scanned Study members did not differ from other living Study members on *p* factor scores, childhood SES, or childhood IQ (see Supplementary Information for details). The relevant ethics committees approved each phase of the Study and informed consent was obtained from all Study members.

### Measuring the general factor of psychopathology, the *p* factor

The Dunedin Study longitudinally ascertains mental disorders every few years, interviewing members about past-year symptoms (see Supplementary Information, including Supplementary Fig. 2, for details). We studied Diagnostic and Statistical Manual of Mental Disorders (DSM)-defined symptoms of the following 14 disorders that were repeatedly assessed in our longitudinal study: ADHD, alcohol dependence, cannabis dependence, dependence on hard drugs, tobacco dependence (assessed with the Fagerström Test for Nicotine Dependence [22]), conduct disorder, major depression, generalized anxiety disorder, fears and/or phobias, eating disorders, PTSD, obsessive compulsive disorder, mania, as well as positive and negative schizophrenia symptoms. Ordinal measures represented the number of possible DSM-defined symptoms associated with each disorder. Fears and/or phobias were assessed as the count of diagnoses for simple phobia, social phobia, agoraphobia, and panic disorder that a Study member reported at each assessment. Each of the 14 disorders was assessed at least 3 times. The past-year prevalence rates of psychiatric disorders in the Dunedin cohort are similar to prevalence rates in nationwide surveys of the United States and New Zealand [23, 24].

The method used to compute a general factor of psychopathology in the Dunedin cohort up to age 38 has been described previously [2]; here we extend these models to include the age-45 data (see Supplementary Information for details). Briefly, we used confirmatory factor analysis to compute a bifactor model specifying a general psychopathology factor (labeled *p*) (Supplementary Fig. 3A, B). In our model, each of the 14 latent disorder factors (defined by assessments across multiple timepoints of the corresponding symptom scale) loaded on the general psychopathology factor (*p*) and cross-loaded on one of three narrower styles of psychopathology. The model also included method/state factors (defined by all symptoms scales within any assessment age), designed to pull out age- and assessment-related variance (e.g., interviewer effects, mood effects) that was uncorrelated with trait propensity toward psychopathology. Data analysis syntax for the model is available at (https://github.com/HaririLab/Publications).

All analyses were performed in Mplus version 8.3 using the weighted least squares means and variance adjusted (WLSMV) algorithm. After respecification for a Heywood case, the bifactor model fit the data well (Supplementary Table 1 and Fig. 3B): *χ*^2^(2457, *n* = 1000) = 3695.364, CFI = 0.949, TLI = 0.945, RMSEA = 0.022, 90% confidence internal (CI) = [0.021, 0.024]. Loadings on the *p* factor were high (all *p*’s < 0.001) and averaged 0.612. For expository purposes, we scaled Study members’ *p* factor scores to *M* = 100, SD = 15. The *p* factor allows us to test for structural brain alterations in relation to general psychopathology. Study members with higher *p* factor scores experienced a greater variety of mental disorders from adolescence to midlife (*r* = 0.77; Supplementary Fig. 4).

### MRI data acquisition

Each study member was scanned using a Siemens Skyra 3T scanner equipped with a 64-channel head/neck coil at the Pacific Radiology imaging center in Dunedin, New Zealand. Diffusion-weighted images providing full brain coverage were acquired with 2.5 mm isotropic resolution and 64 diffusion-weighted directions (4700 ms repetition time, 110.0 ms echo time, *b* value 3000 s/mm^2^, 240 mm field of view, 96 × 96 acquisition matrix, and slice thickness = 2.5 mm). Nonweighted (*b* = 0) images were acquired in both the encoding (AP) and reverse encoding (PA) directions to allow for EPI distortion correction. High-resolution structural images were obtained using a T1-weighted MP-RAGE sequence with the following parameters: TR = 2400 ms; TE = 1.98 ms; 208 sagittal slices; flip angle, 9°; FOV, 224 mm; matrix = 256 × 256; slice thickness = 0.9 mm with no gap (voxel size 0.9 × 0.875 × 0.875 mm); and total scan time = 6 min and 52 s. All neuroimaging data were visually inspected for quality. Data were excluded for Study members who were unable to be scanned with the 64-channel head coil, had an incidental finding, or whose scans were of poor quality due to motion (as revealed by visual inspection for T1-weighted images or >3 mm frame-to-frame movements for diffusion images), resulting in a total of 854 Study members eligible for diffusion analyses and 860 Study members eligible for GMV analyses.

### Fractional anisotropy (FA)

Following the methods of Romer et al. [4], diffusion tensor imaging analyses were completed using SPM8 implemented in Matlab R2016a. All diffusion-weighted scans were motion corrected and co-registered to the mean image to correct for head movement. The tensor model was used to calculate FA values for each voxel and nonbrain tissue was removed. Each image was normalized to Montreal Neurological Institute (MNI) space and smoothed using a 4 mm FWHM Gaussian kernel. We note that the tensor model for derivation of FA values is not optimized for our current diffusion-weighted image data [25], which was acquired with *b* = 3000 s/mm^2^ to facilitate future probabilistic tractography. We are unaware of any suitable alternatives for the derivation of FA values at higher *b* values. Moreover, these differences in acquisition parameters are of less concern because visual inspection of the preprocessed images revealed adequate registration and we did successfully replicate the association between higher *p* factor scores and lower pontine FA (see below).

### Gray matter volume (GMV)

Again, following the methods of Romer et al. [4], regional GMVs were determined using the unified segmentation [26] and DARTEL normalization [27] modules in SPM12 (http://www.fil.ion.ucl.ac.uk/spm). Using this approach, individual T1-weighted images were segmented into gray, white, and CSF images, and then nonlinearly registered to the existing IXI template of 550 healthy subjects averaged in standard MNI space, available with VBM8 (http://dbm.neuro.uni-jena.de/vbm/). Subsequently, gray matter images were modulated for nonlinear effects of the high-dimensional normalization to preserve the total amount of signal from each region and smoothed with an 8 mm FWHM Gaussian kernel. The voxel size of processed images was 1.5 × 1.5 × 1.5 mm. A gray matter mask for subsequent analyses was created by thresholding the final stage (6th) IXI template at 0.1.

### Cerebellar GMV

In addition to the above whole-brain voxel-based GMV analyses, the Spatially Unbiased Infratentorial Toolbox (SUIT) was used for high-resolution cerebellar-specific voxel-based morphometry analyses as per the methods of Romer et al. [4]. For each Study member, the Isolate function of the toolbox was used to create a mask of the cerebellum and generate gray and white matter segmentation maps. The masked segmentation maps were then normalized to the SUIT template with nonlinear DARTEL normalization. The resulting cerebellar gray matter image was resliced into the SUIT atlas space and smoothed with a 4 mm FWHM isotropic Gaussian kernel, a small kernel to preserve precision in the definition of cerebellar structures, in line with previous publications [28].

### ICA of cerebellar morphology

Lastly, we conducted an ICA of SUIT-based cerebellar morphology using the method of Moberget et al. [19]. Briefly, we masked the SUIT-derived cerebellar gray matter maps using the SUIT toolbox's gray matter probability map thresholded at 0.1 and subjected them to ICA using FSL MELODIC [29]. In our sample, a model order of nine corresponded to the highest number of clearly bilateral components, and this model was used for further analyses.

### Statistical analyses

Exact masks were created from the three primary associations with *p* factor scores originally reported in Romer et al. [4]: a 272 voxel cluster in the pons, a 2353 voxel cluster in the visual association cortex, and a 706 voxel cluster in the cerebellum. A fourth mask was created for the 156 voxel cluster in neocerebellar lobule VIIb identified through the SUIT analysis. Moving to the Dunedin Study data, mean values for each of these four masks were extracted for each Study member from the FA (pons), GMV (visual association cortex and neocerebellum), and SUIT maps, respectively. These mean extracted values were then used as the dependent variable in linear models with *p* factor scores as the predictor and sex and total intracranial volume or average total FA, respectively, as covariates to explicitly test for replication of the original findings of Romer et al. [4].

Per the strategy of Moberget et al. [19], we also tested whether weights on our nine ICA-derived cerebellar components could predict *p* factor scores using shrinkage linear regression with 10,000 iterations of tenfold cross-validation on randomly partitioned data. As in Moberget et al. [19], we controlled for sex and total intracranial volume. Performance was evaluated by comparing the distribution of Pearson correlations between predicted and observed *p* factor scores to a null distribution of correlations obtained by randomly permuting the *p* factor scores.

## Results

### White matter microstructural integrity

**Fig. 1:**
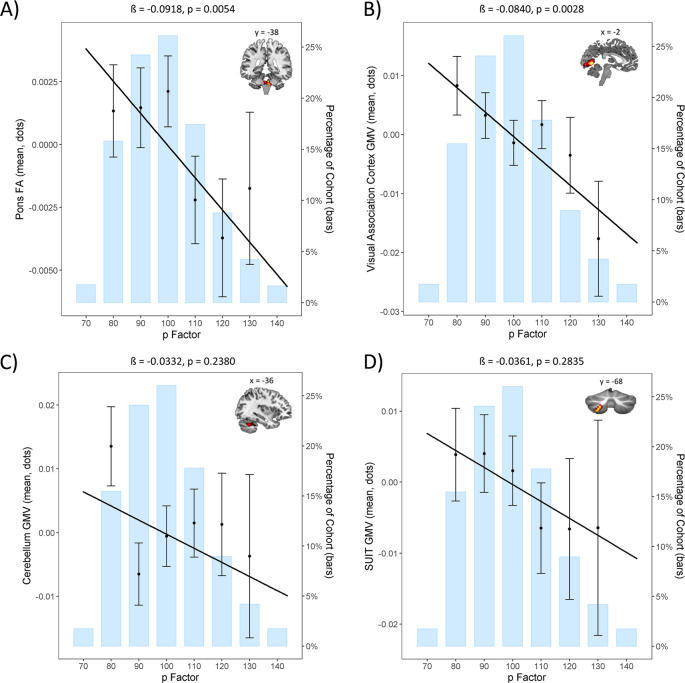
Replication analyses in the Dunedin Study of the original structural brain associations with *p* factor scores from Romer et al. [4]. **a** Replication of the negative association between pontine fractional anisotropy (FA) and *p* factor scores. **b** Replication of the negative association between visual association cortex gray matter volume (GMV) and *p* factor scores. **c** Nonsignificant replication of the negative association between cerebellar GMV and *p* factor scores. **d** Nonsignificant replication of the negative association between SUIT-based neocerebellar lobule VIIb GMV and *p* factor scores. Per convention, *p* factor scores are normalized to a mean of 100 (SD = 15).

A significant negative correlation (standardized *β* = −0.092; *p* = 0.005) indicated an association between lower pontine FA and higher *p* factor scores (Fig. 1a), replicating the finding of Romer et al. [4].

### Gray matter volume (GMV)

A significant negative correlation (standardized *β* = −0.084; *p* = 0.003) indicated an association between decreased visual association cortex GMV and higher *p* factor scores (Fig. 1b), replicating the finding of Romer et al. [4]. An observed negative correlation between cerebellar GMV and *p* factor scores was not statistically significant (standardized *β* = −0.033; *p* = 0.238; Fig. 1c). This was also true for the SUIT-based neocerebellar lobule VIIb cluster (standardized *β* = −0.036; *p* = 0.284; Fig. 1d).

### ICA-derived cerebellar morphology

**Fig. 2:**
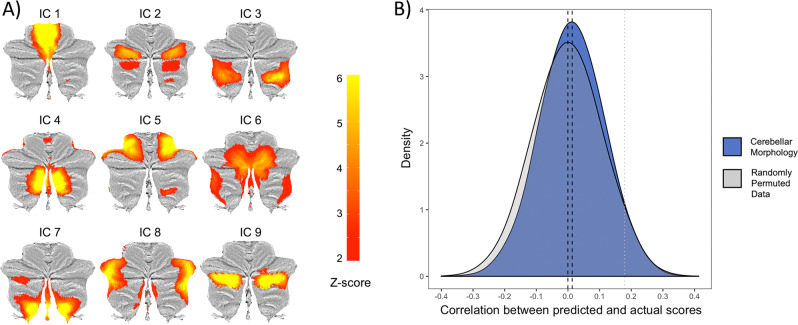
Replication analyses in the Dunedin Study of the original ICA-derived cerebellar morphology associations with *p* factor scores from Moberget et al. [19]. **a** The nine independent components resulting from data-driven decomposition of cerebellar gray matter maps projected onto flat-maps of the cerebellar cortex [36]. **b** Distributions of correlations between predicted and actual *p* factor scores across 10,000 iterations of the tenfold cross-validated model using the average of the nine independent components from **a** compared with the empirical null distribution. The black dotted lines represent the mean for each distribution and the gray dotted line represents the one-tailed 0.05 threshold. The nine ICA-derived components predicted *p* factor scores beyond chance on average, but the difference from the empirical null distribution was *p* = 0.53, suggesting nonsignificant replication of Moberget et al. [19].

The nine independent components of cerebellar morphology collectively accounted for 41.47% of the total variance in the modulated gray matter maps; each component explained between 4.08 and 4.97% of the total variance (and between 9.83 and 11.99% of the explained variance). The nine ICA-derived components predicted *p* factor scores beyond chance on average, but the difference from the empirical null distribution was not significant (mean correlations between predicted and observed values: *r* = 0.13, *p* = 0.53; mean *r* > 54.89% of the empirical null distribution; Fig. 2).

## Discussion

We successfully replicated two prior associations between variation in brain structure and general psychopathology, as indexed by the *p* factor, using data from a population-representative birth cohort now in midlife. Namely, we replicated associations between *p* factor scores and both pontine FA and visual association cortex GMV as originally reported by Romer et al. [4]. In contrast, we failed to replicate three prior associations between cerebellar structure and *p* factor scores. First, although nominally consistent with the original report of Romer et al. [4], neither of two tested associations between GMV in a broad cerebellar cluster nor a smaller cluster in neocerebellar lobule VIIb were statistically significant. Second, an ICA-based measure of global cerebellar morphology did not significantly predict *p* factor scores above chance as was reported originally by Moberget et al. [19].

The replication of a negative association between pontine FA and *p* factor scores further implicates the CTCC in general psychopathology. Thus, dysfunction in fundamental aspects of monitoring and coordinating executive functions (i.e., “forward control”) through dynamic information processing between the neocerebellum and prefrontal cortex appears to be a core transdiagnostic feature of general psychopathology. The second replication of a negative association between *p* factor scores and GMV in visual association cortex is consistent with the importance of executive dysfunction in general psychopathology. In particular, structural alterations in visual association cortex may manifest as more effortful or less efficient integration of bottom-up sensory information with attentional demands and executive control processes in individuals who meet criteria for different forms of mental disorders [20].

The nonsignificant associations between *p* factor scores and multiple indices of cerebellar GMV and morphology do not necessarily undermine the importance of a prefrontal CTCC in general psychopathology. Rather, these failures may indicate that shared variation among different forms of psychopathology, as captured by the *p* factor, is more a reflection of how information is communicated within the CTCC, particularly through pontine white matter pathways connecting the prefrontal cortex and cerebellum, and less a reflection of how information may be locally computed within the neocerebellum. This would be consistent with the emerging understanding that brain function may be best characterized by distributed patterns of network communication rather than discrete regional activity [30, 31]. However, there also are pragmatic factors that may have limited our ability to replicate prior associations between *p* factor scores and cerebellar structure.

First, the failure to replicate cerebellar associations with *p* factor scores may reflect different contributions of brain structure to risk across development. The discovery samples were comprised of young adults [4] or children, adolescents, and young adults [19]. In contrast, our sample is comprised of individuals in midlife. Thus, the contribution of cerebellar GMV and morphology to the *p* factor may be greater earlier than later in life. This difference may reflect the still-active structural development of the cerebellum, which parallels that of the prefrontal cortex, in both discovery samples [32]. Developmental differences are hinted at by the observation of only nine independent components of cerebellar morphology in our sample but ten such components in the sample studied by Moberget et al. [19]. Longitudinal assessment of brain structure and *p* factor scores within the same individuals is necessary to evaluate a hypothesis of developmental differences [33]. Second, the nature of the sampling strategy across the three samples also may influence replication. Unlike the population-representative birth cohort in our current study, both discovery samples represented narrow groups of select individuals (e.g., high-functioning university students or community volunteers). Additional replication efforts across diverse samples are necessary to probe the implications of such possible differences for the study of the brain basis of general psychopathology. Lastly, we may simply have been underpowered to identify significant associations of small effect. Our current sample is smaller than either the discovery sample of Romer et al. [4] (*N* = 1246) or Moberget et al. [19] (*N* = 1401). Generally, successful replication is more likely if the test samples are larger and thus better powered to detect often smaller effects than reported in a discovery sample [34, 35]. The effect sizes in Romer et al. [4] ranged from *r* = 0.09 to 0.13 and those in Moberget et al. [19] ranged from *r* = 0.13 to 0.2.

These limitations notwithstanding, the two replicable associations of the theory-free, data-driven findings of Romer et al. [4] reported herein point to specific features of brain structure that may be a core feature of shared variation among common forms of mental illness. Alterations in the microstructural integrity of pontine white matter pathways may reflect dysfunction of executive control processes supported through dynamic communication within the CTCC. Likewise, alterations in GMV of visual association cortex may reflect impairments in the integration of bottom-up sensory information with top-down executive control and attentional processes. Notably, both of these neuroanatomical features are consistent with a model of the *p* factor as indexing increasingly disordered thought, which characterizes the most debilitating forms of mental disorders. The extent to which these neuroanatomical features drive the emergence of general psychopathology or emerge as a consequence of general psychopathology are as yet unknown and require longitudinal neuroimaging assessments to explicate.

## Supplementary information

Supplementary Information

## References

[1] Lahey BB, Krueger RF, Rathouz PJ, Waldman ID, Zald DH (2017). A hierarchical causal taxonomy of psychopathology across the life span. Psychol Bull.

[2] Caspi A, Houts RM, Belsky DW, Goldman-Mellor SJ, Harrington H, Israel S (2014). The p factor: One general psychopathology factor in the structure of psychiatric disorders?. Clin Psychol Sci J Assoc Psychol Sci.

[3] Caspi A, Moffitt TE (2018). All for one and one for all: mental disorders in one dimension. Am J Psychiatry.

[4] Romer AL, Knodt AR, Houts R, Brigidi BD, Moffitt TE, Caspi A (2018). Structural alterations within cerebellar circuitry are associated with general liability for common mental disorders. Mol Psychiatry.

[5] Buckner RL, Krienen FM, Castellanos A, Diaz JC, Yeo BTT (2011). The organization of the human cerebellum estimated by intrinsic functional connectivity. J Neurophysiol.

[6] Thomas Yeo BT, Krienen FM, Sepulcre J, Sabuncu MR, Lashkari D, Hollinshead M (2011). The organization of the human cerebral cortex estimated by intrinsic functional connectivity. J Neurophysiol.

[7] Middleton FA, Strick PL (2001). Cerebellar projections to the prefrontal cortex of the primate. J Neurosci.

[8] Buckner RL (2013). The cerebellum and cognitive function: 25 years of insight from anatomy and neuroimaging. Neuron.

[9] D’Angelo E, Casali S. Seeking a unified framework for cerebellar function and dysfunction: from circuit operations to cognition. Front Neural Circuits. 2013;6. https://www.frontiersin.org/articles/10.3389/fncir.2012.00116/full#F3. 10.3389/fncir.2012.00116 PMC3541516 23335884

[10] Keren-Happuch E, Chen S-HA, Ho M-HR, Desmond JE (2014). A meta-analysis of cerebellar contributions to higher cognition from PET and fMRI studies. Hum Brain Mapp.

[11] Marek S, Siegel JS, Gordon EM, Raut RV, Gratton C, Newbold DJ (2018). Spatial and temporal organization of the individual human cerebellum. Neuron.

[12] Ito M (1993). Movement and thought: identical control mechanisms by the cerebellum. Trends Neurosci.

[13] Ito M (2008). Control of mental activities by internal models in the cerebellum. Nat Rev Neurosci.

[14] Bernard JA, Mittal VA (2015). Dysfunctional activation of the cerebellum in schizophrenia: a functional neuroimaging meta-analysis. Clin Psychol Sci J Assoc Psychol Sci.

[15] Moberget T, Doan NT, Alnæs D, Kaufmann T, Córdova-Palomera A, Lagerberg TV (2018). Cerebellar volume and cerebellocerebral structural covariance in schizophrenia: a multisite mega-analysis of 983 patients and 1349 healthy controls. Mol Psychiatry.

[16] Andreasen NC, Paradiso S, O’Leary DS (1998). “Cognitive Dysmetria” as an integrative theory of schizophrenia: a dysfunction in cortical-subcortical-cerebellar circuitry?. Schizophr Bull.

[17] Schmahmann JD (2004). Disorders of the cerebellum: Ataxia, dysmetria of thought, and the cerebellar cognitive affective syndrome. J Neuropsychiatry Clin Neurosci.

[18] Schmahmann JD, Weilburg JB, Sherman JC (2007). The neuropsychiatry of the cerebellum—insights from the clinic. Cerebellum.

[19] Moberget T, Alnæs D, Kaufmann T, Doan NT, Córdova-Palomera A, Norbom LB (2019). Cerebellar gray matter volume is associated with cognitive function and psychopathology in adolescence. Biol Psychiatry.

[20] Elliott ML, Romer A, Knodt AR, Hariri AR (2018). A connectome-wide functional signature of transdiagnostic risk for mental illness. Biol Psychiatry.

[21] Poulton R, Moffitt TE, Silva PA (2015). The Dunedin Multidisciplinary Health and Development Study: Overview of the first 40 years, with an eye to the future. Soc Psychiatry Psychiatr Epidemiol.

[22] Heatherton TF, Kozlowski LT, Frecker RC, Fagerström KO (1991). The Fagerström test for nicotine dependence: a revision of the Fagerström Tolerance Questionnaire. Br J Addict.

[23] Schaefer JD, Caspi A, Belsky DW, Harrington H, Houts R, Horwood LJ (2017). Enduring mental health: prevalence and prediction. J Abnorm Psychol.

[24] Moffitt TE, Caspi A, Taylor A, Kokaua J, Milne BJ, Polanczyk G (2010). How common are common mental disorders? Evidence that lifetime prevalence rates are doubled by prospective versus retrospective ascertainment. Psychol Med.

[25] Steven AJ, Zhuo J, Melhem ER (2013). Diffusion Kurtosis imaging: an emerging technique for evaluating the microstructural environment of the brain. Am J Roentgenol.

[26] Ashburner J, Friston KJ (2005). Unified segmentation. NeuroImage..

[27] Ashburner J (2007). A fast diffeomorphic image registration algorithm. NeuroImage..

[28] D’Agata F, Caroppo P, Boghi A, Coriasco M, Caglio M, Baudino B (2011). Linking coordinative and executive dysfunctions to atrophy in spinocerebellar ataxia 2 patients. Brain Struct Funct.

[29] Beckmann CF, Smith SM (2004). Probabilistic independent component analysis for functional magnetic resonance imaging. IEEE Trans Med Imaging.

[30] Mišić B, Sporns O (2016). From regions to connections and networks: new bridges between brain and behavior. Curr Opin Neurobiol.

[31] Bassett DS, Sporns O (2017). Network neuroscience. Nat Neurosci.

[32] Diamond A (2000). Close interrelation of motor development and cognitive development and of the cerebellum and prefrontal cortex. Child Dev.

[33] Schaie K (1967). Age changes and age differences. Gerontologist..

[34] Anderson SF, Maxwell SE (2017). Addressing the “Replication Crisis”: using original studies to design replication studies with appropriate statistical power. Multivar Behav Res.

[35] Turner BO, Paul EJ, Miller MB, Barbey AK (2018). Small sample sizes reduce the replicability of task-based fMRI studies. Commun Biol.

[36] Diedrichsen J, Zotow E (2015). Surface-based display of volume-averaged cerebellar imaging data. PLoS One.

